# Small-Leaved Kuding Tea Extract Fractions Attenuate Hyperlipidemia and Hepatic Steatosis in Mice: Association with Upregulation of Fatty Acid β-Oxidation Enzymes and Improvement of Metabolic Biomarkers

**DOI:** 10.3390/nu18203345

**Published:** 2026-10-13

**Authors:** Ting Ye, Jia-Hui Li, Qin-Qin Xiong, Ming-Juan Chen, Sha Cheng, Li-Rong Huang, Heng Luo

**Affiliations:** 1 State Key Laboratory of Discovery and Utilization of Functiona Components in Traditional Chinese Medicine, Natural Products Research Center of Guizhou Province, Guizhou Medical University, Guiyang 550014, China; 2 School of Biological and Environmental Engineering, Guiyang University, Guiyang 550005, China; 3 College of Food and Pharmaceutical Engineering, Guizhou Institute of Technology, Guiyang 550025, China

**Keywords:** hyperlipidemia, small-leaved kuding tea (SLKT), fatty acid degradation, lipid metabolism, oxidative stress, ACADM/EHHADH/HADHB

## Abstract

Background/Objectives: Hyperlipidemia is a prevalent metabolic disorder that increases cardiovascular disease risk and is associated with lipid accumulation, inflammation, and oxidative stress. Small-leaved Kuding tea (SLKT) has multiple health benefits, but its lipid-lowering mechanisms remain unclear. This study evaluated the therapeutic effects of different SLKT fractions on hyperlipidemia and investigated their modulation of the fatty acid degradation pathway. Methods: A hyperlipidemia mouse model was established using a high-fat diet for 8 weeks. Sixty successfully modeled mice were randomly divided into six groups: model control, anhydrous ethanol fraction (SLK-AE), ethyl acetate fraction (SLK-EA), n-butanol fraction (SLK-NA), water-soluble fraction (SLK-WA), and simvastatin (positive control). Mice received daily gavage for 8 weeks. Serum biochemical indices, inflammatory factors, hepatic oxidative stress markers, and liver histopathology were assessed. Protein expression levels of ACADM, EHHADH, and HADHB in the liver were detected by Western blot. Results: SLKT fractions significantly reduced body weight, fat mass, serum TC, TG, LDL-C, Glu, NEFA, ALT, AST, TNF-α, IL-1β, IL-6, and LPS, while increasing HDL-C (*p* < 0.05). Hepatic TC, TG, ALT, AST, and MDA decreased, and SOD increased (*p* < 0.05). Histological staining revealed markedly reduced lipid droplets in hepatocytes. Furthermore, SLKT significantly upregulated hepatic protein expression of ACADM, EHHADH, and HADHB, key enzymes in fatty acid β-oxidation (*p* < 0.05). Conclusions: SLKT effectively ameliorates hyperlipidemia by improving lipid profiles, reducing serum biomarkers of liver injury and pro-inflammatory cytokines, and upregulating key fatty acid degradation enzymes. These findings support SLKT as a potential natural therapeutic agent for hyperlipidemia and associated metabolic disorders.

## 1. Introduction

Hyperlipidemia is a prevalent metabolic disorder characterized by dysregulation in the transport and metabolism of plasma lipids, leading to elevated levels of total cholesterol (TC), triglycerides (TG), and low-density lipoprotein cholesterol (LDL-C), often accompanied by reduced high-density lipoprotein cholesterol (HDL-C) [1]. This lipid metabolism disorder not only increases the risk of cardiovascular diseases but also results in lipid accumulation in blood vessel walls. Consequently, this accumulation can reduce vascular elasticity, cause vessel wall hardening and stenosis, and potentially lead to persistent constriction of the coronary arteries and vascular sclerosis [2]. Furthermore, lipids transported to the liver via the bloodstream can accumulate in hepatocytes, triggering metabolic abnormalities that further exacerbate the risk of cardiovascular diseases [3].

The global burden of hyperlipidemia has reached alarming proportions. According to the China Cardiovascular Health and Disease Report 2023, released by the Health Planning Commission in 2024, cardiovascular diseases are the leading cause of death among urban and rural residents in China, surpassing tumors and posing a significant threat to human health [4]. Epidemiological data indicate that dyslipidemia affects approximately 19% of Chinese children and adolescents, with prevalence increasing to 27% among those aged 12–18 years. Among hypertensive patients, the comorbidity rate of dyslipidemia reaches 37.5%, substantially elevating cardiovascular event risks. Therefore, reducing blood lipid levels and improving dyslipidemia metabolism are critical measures to decrease the morbidity and mortality of cardiovascular and cerebrovascular diseases. Therefore, reducing blood lipid levels and improving dyslipidemia metabolism are critical measures to decrease the morbidity and mortality of cardiovascular and cerebrovascular diseases.

Hepatic lipid homeostasis is maintained through a delicate balance among lipid uptake, de novo lipogenesis, fatty acid oxidation, and very low-density lipoprotein (VLDL) secretion. Fatty acid β-oxidation is a central catabolic pathway that breaks down long-chain fatty acids into acetyl-CoA units, which enter the tricarboxylic acid cycle for ATP production or are converted into ketone bodies. The β-oxidation spiral consists of four sequential enzymatic reactions: (1) dehydrogenation by acyl-CoA dehydrogenases (ACADs), (2) hydration by enoyl-CoA hydratase, (3) a second dehydrogenation by 3-hydroxyacyl-CoA dehydrogenase, and (4) thiolytic cleavage by 3-ketoacyl-CoA thiolase. Dysregulation of this pathway leads to excessive lipid accumulation in hepatocytes, contributing to hepatic steatosis and insulin resistance [5,6].

In the present study, we focused on three key enzymes positioned at different stages of the β-oxidation cascade. ACADM (acyl-CoA dehydrogenase medium chain) catalyzes the initial dehydrogenation of medium-chain acyl-CoA substrates and serves as a rate-limiting enzyme in the β-oxidation pathway; its deficiency has been linked to metabolic inflexibility and hepatic lipid accumulation [7]. EHHADH (enoyl-CoA hydratase and 3-hydroxyacyl-CoA dehydrogenase) is a bifunctional peroxisomal enzyme that catalyzes both the hydration and the second dehydrogenation steps, playing a critical role in very long-chain fatty acid oxidation [8]. HADHB (hydroxyacyl-CoA dehydrogenase trifunctional multienzyme complex subunit beta) is the beta subunit of the mitochondrial trifunctional protein, which catalyzes the final three steps (hydration, dehydrogenation, and thiolysis) of long-chain fatty acid β-oxidation. Together, these three enzymes cover the initiation (ACADM), middle (EHHADH), and terminal (HADHB) stages of the β-oxidation pathway, providing a comprehensive assessment of fatty acid oxidative capacity. Dysregulation of each of these enzymes has been associated with metabolic disorders.

In recent years, natural products have attracted considerable attention as potential therapeutic agents for hyperlipidemia due to their multi-target characteristics and favorable safety profiles. For example, resveratrol has been shown to activate AMPK and SIRT1, thereby promoting fatty acid oxidation and reducing hepatic steatosis [9]; curcumin exerts lipid-lowering effects through modulation of PPAR-γ and suppression of pro-inflammatory cytokines [10]; and green tea extract increases adiponectin and PPAR α levels to improve hepatic steatosis [11]. These findings underscore the potential of plant-derived polyphenols in managing metabolic disorders and provide a rationale for investigating the lipid-lowering mechanisms of small-leaved Kuding tea extracts (SLKTEs).

Currently, the clinical treatment of hyperlipidemia primarily relies on chemical drugs, such as statins, lipid-lowering agents, intestinal cholesterol absorption inhibitors, fibrates, niacin and its derivatives, and resins [12]. These drugs alleviate dyslipidemia by reducing cholesterol uptake or inhibiting synthesis pathways, thereby lowering blood lipid levels [13]. However, despite their widespread use due to well-defined mechanisms of action, these chemical drugs are associated with numerous adverse effects, which limit their suitability for long-term clinical use [14]. Additionally, the complex etiology of hyperlipidemia and the diversity of clinical symptoms make it challenging for a single drug to achieve significant therapeutic efficacy. Consequently, the discovery of novel multi-pathway and multi-system lipid-lowering drugs is of paramount importance.

Small-leaved Kuding tea (SLKT), known for its multiple health benefits due to its unique medicinal and food properties, has been traditionally used for clearing heat, detoxifying, calming effects, quenching thirst, promoting digestion, expectorant properties, regulating liver function, and improving vision [15,16,17]. Modern medical research has further confirmed that SLKT possesses health-promoting functions, including anti-inflammatory, antiseptic, antioxidant, weight loss, and diuretic effects, making it highly popular among the public [18,19,20]. Studies have also shown that long-term consumption of SLKT is associated with a lower incidence of hypertension and cardiovascular diseases, suggesting its potential value in the prevention of cardiovascular conditions [21]. Therefore, investigating how SLKT treats hyperlipidemia by modulating the fatty acid degradation pathway can provide a scientific basis for its potential as a natural lipid-lowering agent and contribute to the development of new strategies for the prevention and treatment of hyperlipidemia [22].

In the present study, a mouse model of hyperlipidemia was successfully established, and different fractions of SLKT (anhydrous ethanol fraction, ethyl acetate fraction, n-butanol fraction, and water-soluble fraction) were administered to the model animals. The lipid-lowering, anti-inflammatory, antioxidant, and hepatoprotective effects of SLKT were systematically evaluated. The results of this study provide new insights into the prevention and treatment of hyperlipidemia and offer an experimental basis for the use of SLKT as a potential natural hepatoprotective agent.

## 2. Materials and Methods

### 2.1. Materials

#### 2.1.1. Laboratory Animals

Eighty 5-week-old male C57BL/6J mice, each weighing approximately 20 g, were used in this study. Approval was obtained from the ethics committee of Guizhou Medical University (1901110). The experiments were conducted under Animal Production License No. SYXK (Guizhou) 2018-0001. Upon arrival, the animals were individually housed in cages located in the SPF-grade animal room at the Guizhou Natural Products Research Center, with 10 mice per cage. The room temperature was meticulously maintained at 22.0 ± 2.0 °C, and the relative humidity was controlled at 55.0 ± 10%. Additionally, a natural circadian light cycle was provided to ensure optimal physiological conditions. Throughout the study, the mice had unrestricted access to both feed and water, ensuring their nutritional and hydration needs were consistently met.

#### 2.1.2. Drugs and Reagents

SLKT (Guizhou Taihe Modern Eco-Agriculture Science and Technology Co., Ltd, Zunyi, China). High-fat diet containing 60 kcal% fat (Shuyu Biotechnology Co., Ltd., Shanghai, China). TC assay kit (Shenzhen Rayto Life and Analytical Sciences Co., Ltd., Shenzhen, China), Lot No. 20230608; TG assay kit (Shenzhen Rayto Life and Analytical Sciences Co., Ltd., Shenzhen, China), Lot No. 20230607; HDL-C assay kit (Shenzhen Rayto Life and Analytical Sciences Co., Ltd., Shenzhen, China), Lot No. 20230625; LDL-C assay kit (Shenzhen Rayto Life and Analytical Sciences Co., Ltd., Shenzhen, China), Lot No. 20230605; NEFA (non-esterified fatty acids) assay kit (Shenzhen Rayto Life and Analytical Sciences Co., Ltd., Shenzhen, China), Lot No. 20230608; ALT (Alanine Aminotransferase) assay kit (Shenzhen Rayto Life and Analytical Sciences Co., Ltd., Shenzhen, China), Lot No. 20230706; AST (Aspartate Aminotransferase) assay kit (Shenzhen Rayto Life and Analytical Sciences Co., Ltd., Shenzhen, China), Lot No. 20230707; GLU (Glucose), Lot No. 20230606; TBA (Total Bile Acid) assay kit (Shenzhen Rayto Life and Analytical Sciences Co., Ltd., Shenzhen, China), Lot No. S03074. SOD (superoxide dismutase) assay kit (Wuhan Servicebio Technology Co., Ltd., Wuhan, China), Lot No. MPC2309001; MDA (malondialdehyde) assay kit (Wuhan Servicebio Technology Co., Ltd., Wuhan, China), Lot No. MPC2303081; BCA protein assay kit (Wuhan Servicebio Technology Co., Ltd., Wuhan, China), Lot No. CR2203100-1; IL-1β (Interleukin-1 Beta) assay kit (Wuhan Servicebio Technology Co., Ltd., Wuhan, China), Lot No. RRDC2401015; TNF-α (Tumor Necrosis Factor Alpha) assay kit (Wuhan Servicebio Technology Co., Ltd., Wuhan, China), Lot No. RRDC2401010. IL-6 (Interleukin-6) assay kit (Thermo Fisher Scientific (China) Co., Ltd., Shanghai, China), Lot No. 388298-001. LPS (lipopolysaccharide) assay kit (USCN KIT INC., Wuhan, China), Lot No. L231116870. Simvastatin (Zhejiang Jingxin Pharmaceutical Co., Ltd., Shaoxing, China).

#### 2.1.3. Main Instruments

A high-speed tissue grinder, palm centrifuge, and vortex mixer were provided by Wuhan Xavier Biotechnology Co., Ltd. (Wuhan, China); an enzyme labeling detector by BioTek (Winooski, VT, USA); a benchtop high-speed cryo-centrifuge by Dalian Dahlong Biotechnology Co., Ltd. (Dalian, China); a fully automated biochemistry analyzer and fully automated plate washer by Shenzhen Radu Life Science Co., Ltd. (Shenzhen, China); and a multi-color fluorescence biomolecular imaging system by Bio-Rad Laboratories (Hercules, CA, USA).

### 2.2. Methods

#### 2.2.1. Pharmaceutical Preparation

High-quality, dry SLKT leaves (3 kg) were accurately weighed and crushed through a 60-mesh sieve. The powdered leaves were mixed with distilled water and subjected to extraction with hot water at 65 °C for 30 min, followed by ultrasonic vibration-assisted extraction for an additional 30 min. This two-step extraction process was repeated three times to ensure exhaustive extraction. The resulting filtrates were pooled and filtered through a 0.45 μm membrane to remove residual particulate matter, and the combined filtrate was concentrated under reduced pressure to obtain the crude aqueous extract.

The SLK aqueous extract concentrate was extracted with equal volumes of petroleum ether, ethyl acetate, and n-butanol sequentially, yielding the petroleum ether fraction, the ethyl acetate fraction (SLK-EA), and the n-butanol fraction (SLK-NA), respectively. The residual aqueous phase after these three successive liquid–liquid extractions was defined as the water-soluble fraction (SLK-WA). SLK-EA, SLK-NA, and SLK-WA were obtained from materials prepared in our previous study [23].

In contrast, the anhydrous ethanol fraction (SLK-AE) was separately prepared in the present study and was not derived from the same batch of starting material described in the previous study [23]. Briefly, the concentrated aqueous extract was directly treated with an equal volume of anhydrous ethanol and kept at 4 °C overnight to precipitate polysaccharides and other water-soluble macromolecular components. The supernatant was collected and evaporated under reduced pressure to obtain SLK-AE.

All fractions (SLK-EA, SLK-NA, and SLK-AE) were completely evaporated to dryness under reduced pressure, followed by freeze-drying. SLK-WA was lyophilized directly from the residual aqueous phase. The dried samples were stored at −20 °C for further use.

#### 2.2.2. Group Modeling and Administration of Experimental Animals

Eighty 5-week-old male C57BL/6J mice were acclimatized and fed for one week to ensure they adapted to the laboratory environment. Subsequently, these 80 mice were divided into 10 normal control mice and 70 experimental mice using a computerized random number table. The control group was fed a standard normal diet daily, while the experimental group was fed a high-fat diet daily to establish a hyperlipidemia animal model. After 8 weeks of high-fat diet feeding, serum samples were randomly collected from the mice to measure TC, TG, HDL-C, LDL-C, and Glu. Additionally, abdominal fat and epididymal fat were collected and weighed. The hyperlipidemia mouse model was considered successfully established when the TC, TG, and LDL-C levels in the model group were significantly higher than those in the normal group (*p* < 0.05).

Sixty successfully modeled mice in good condition were then randomly divided into six groups: a model control group, an anhydrous ethanol component administration group (SLK-AE), an ethyl acetate component administration group (SLK-EA), an n-butanol component administration group (SLK-NA), a water-soluble component administration group (SLK-WA), and a simvastatin group (SV); SLKTE was dissolved in physiological saline solution in all groups. The same batch of fractions was used throughout all animal experiments in this study. The fractions subjected to in vivo evaluation were SLK-AE, SLK-EA, SLK-NA, and SLK-WA. Daily gavage administration was performed at 9:00 AM. The control and high-fat diet groups received saline (0.1 mL/10 g/d), the drug administration groups received their respective components (500 mg/kg/d), and the positive control group received simvastatin (2 mg/kg/d). The gavage administration continued for 8 weeks. During the experimental period, general signs, diet, body weight changes, and mortality were closely monitored. After 8 weeks of administration, blood was collected from the orbital venous plexus of the mice and centrifuged at 3000 rpm for 15 min to separate the serum. The serum samples were individually labeled and stored in an ultra-low-temperature freezer at −80 °C. The liver, abdominal white fat, and epididymal adipose tissue were extracted and weighed. Liver lobes were placed in sterilized cryotubes, flash-frozen in liquid nitrogen, and stored in a −80 °C freezer for further analysis.

#### 2.2.3. Detection of Serum Biochemical Indexes and Inflammatory Factor Levels

The serum levels of TC, TG, HDL-C, LDL-C, Glu, and NEFA in each group of mice were measured using specific enzymatic methods. Specifically, the CHOD-PAP (Cholesterol Oxidase-Peroxidase Coupled Method) method was employed for TC, the GPO-PAP (Glycerol-3-Phosphate Oxidase-Peroxidase Coupled Method) method for TG, the direct method-selective inhibition method for HDL-C, the direct method-surfactant scavenging method for LDL-C, the glucose oxidase method for Glu, and the FAO-PAP method for NEFA. Additionally, the levels of AST and ALT in the serum were detected using the aspartate substrate method and the alanine substrate method, respectively. Furthermore, the serum levels of inflammatory factors, including TNF-α, IL-1β, IL-6, and LPS, were measured according to the instructions provided with the ELISA kits.

#### 2.2.4. Detection of Liver Biochemical Indicators and Oxidative Stress Factor Levels

An appropriate amount of liver tissue was weighed, and saline was added at a ratio of weight (g) to volume (mL) = 1:9. The tissue was homogenized mechanically under ice-water bath conditions to prepare a 10% homogenate. The homogenate was then centrifuged at 2500 r/min for 15 min at 4 °C, and the supernatant was collected. The levels of TC, TG, AST, and ALT in the liver tissues of each group were measured using the CHOD-PAP, GPO-PAP, aspartate substrate, and alanine substrate methods, respectively.

The levels of SOD, MDA, and TBA in the liver were determined using commercial assay kits. For SOD activity measurement, the reaction buffer, substrate solution, and chromogenic agent were prepared according to the manufacturer’s instructions. In a 96-well plate, 20 μL of tissue supernatant, 200 μL of reaction buffer, and 20 μL of substrate solution were added sequentially and mixed thoroughly, and then the chromogenic agent was added. After incubation at 37 °C for 20 min, the optical density (OD) was measured at 450 nm using a microplate reader, and SOD activity was calculated. For MDA determination, 100 μL of tissue supernatant and 200 μL of TBA solution were mixed in a centrifuge tube and incubated in a 95 °C water bath for 40 min. The reaction was terminated by cooling on ice, followed by centrifugation at 3000 rpm for 15 min. The OD of the supernatant was measured at 532 nm, and MDA content was calculated. For TBA measurement, 10 μL of tissue supernatant, 150 μL of reaction buffer, and 40 μL of enzyme working solution were added to a 96-well plate, mixed thoroughly, and incubated at 37 °C for 10 min. The OD was measured at 405 nm, and TBA content was calculated. Protein concentrations in tissue supernatants were determined using a BCA assay: 25 μL of tissue supernatant and 200 μL of BCA working solution were mixed in a 96-well plate and incubated at room temperature for 30 min, after which the OD was measured at 562 nm. All measured SOD, MDA, and TBA values were normalized to the corresponding protein concentrations.

#### 2.2.5. Detection of Liver Tissue Morphology

Fresh liver tissues from mice (approximately 0.5 cm^2^) were fixed in 4% paraformaldehyde for 48 h, routinely embedded in paraffin, and sectioned into 4 μm slices. The sections were then stained with H&E. Simultaneously, paraffin-embedded liver tissues were sectioned into approximately 10 μm slices, stained with Oil Red O staining solution for 8–10 min, differentiated twice with 60% isopropyl alcohol, and rinsed twice. The sections were counterstained with hematoxylin for 3–5 min. Changes in cell morphology were observed under a light microscope at 400× magnification.

#### 2.2.6. Protein Expression Levels of ACADM, EHHADH, and HADHB in the Liver

Frozen liver tissues from the control group, high-fat diet group, SLK-AE group, SLK-EA group, SLK-NA group, and SV group were selected. Tissue proteins were extracted, and the protein concentration was determined using the BCA protein quantification method. The proteins were separated by SDS-PAGE gel electrophoresis and transferred to a PVDF membrane. After blocking for 1 h, the membrane was incubated with the primary antibody at 4 °C overnight, followed by washing with TBST. The membrane was then incubated with the secondary antibody for 1 h at room temperature and washed three times with TBST. The membrane was exposed using an imaging system, and the image was developed and photographed. The bands were analyzed and decolorized using Alpha software 4.0.0. The optical density of each band was calculated, and the target proteins were semi-quantitatively analyzed using α-tubulin as the internal reference.

### 2.3. Statistical Analysis

Experimental data are expressed as mean ± SD. Statistical analysis was performed using SPSS 26.0 and GraphPad Prism 8.0.2 statistical analysis software. Comparisons between two groups were conducted using *t*-tests, and comparisons between multiple groups were made using one-way ANOVA. *p* < 0.05 indicated that the differences were statistically significant.

## 3. Results

### 3.1. Determination of Chemical Constituents of SLKTEs

Compositional analysis was performed on SLK-PE, SLK-EA, SLK-NA and SLK-AE. The chromatographic analysis results for SLK-PE, SLK-EA and SLK-NA fractions were referenced to our previous study [23]. LC-MS/MS analysis data of SLK-AE fraction are summarized in Supplementary Tables S1 and S2. Importantly, SLK-AE was prepared via ethanol precipitation from an independent batch of starting material rather than the sequential partitioning workflow described in Ref. [23]; accordingly, compositional profiles reported in Ref. [23] cannot be extrapolated to SLK-AE.

Among the detected compounds, rhoifolin, poliumoside, apigenin-7-O-β-D-glucoside, and rutin exhibited the largest peak areas, suggesting that these compounds may serve as the principal bioactive constituents of SLKT. In addition to these major constituents, the SLKTE fraction also contained other flavonoids and flavonoid glycosides, such as luteolin, apigenin, kaempferol, isoquercitrin, cynaroside, lonicerin, verbenalin, vicenin II, kaempferol-3-O-rutinoside, kaempferol-7-O-β-D-glucopyranoside, and apigenin-7-O-β-D-glucoside; phenylethanoid glycosides, including verbascoside, plantamajoside, purpureaside C, and poliumoside; phenolic acids and phenolic alcohols, such as 3,4-dihydroxyphenylethanol, caffeic acid, protocatechualdehyde, salidroside, oleuropein, *p*-hydroxy-cinnamic acid, quinic acid, and orsellinic acid; coumarins, such as coumarin and esculetin; and nucleosides, amino acids, and sugar alcohols, including adenine, adenosine, guanine, guanosine, lactose, and mannitol.

### 3.2. Effect of SLKTE on High-Fat-Diet-Induced Obesity

**Figure 1:**
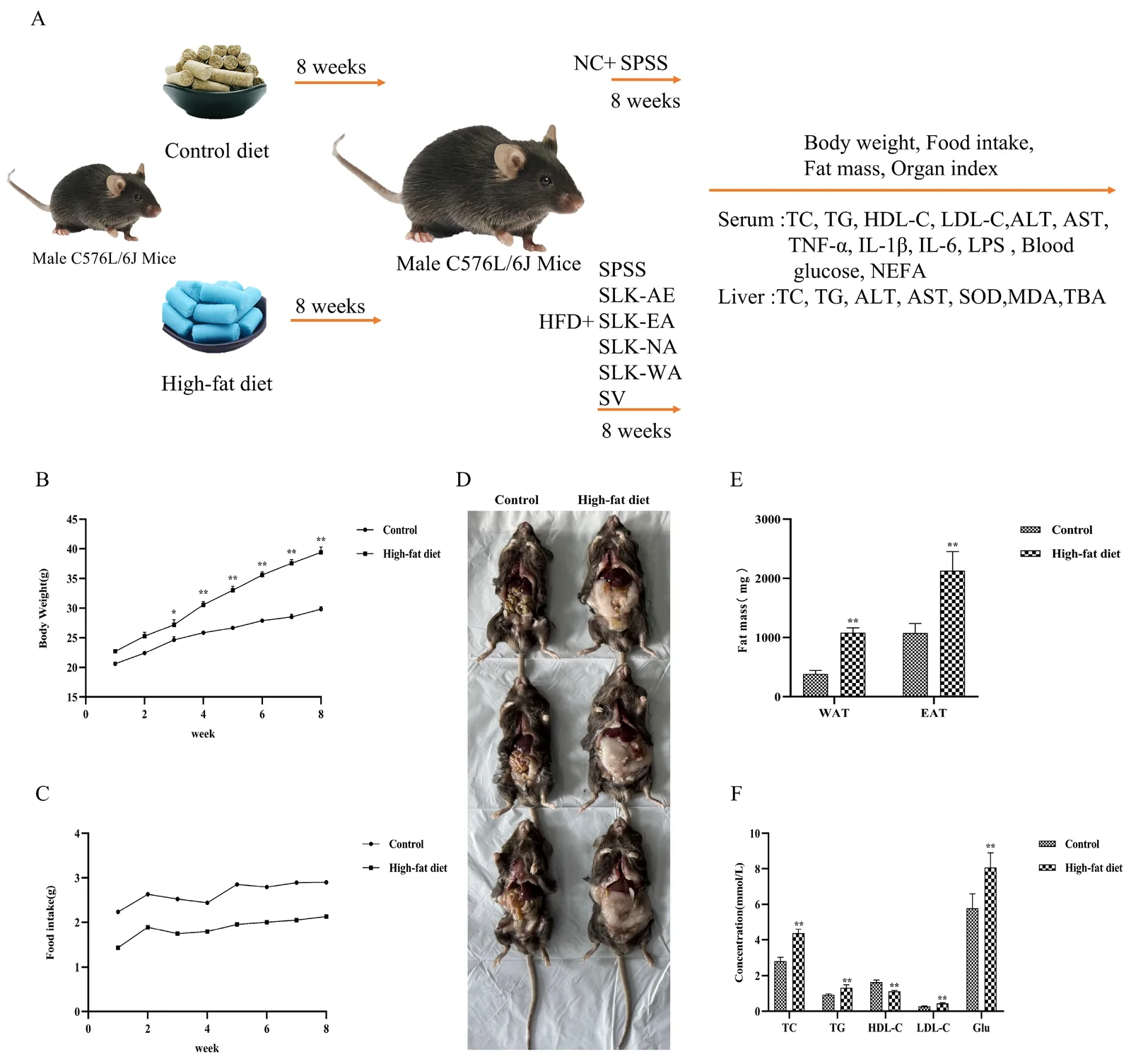
Mice fed a high-fat diet for eight weeks developed hyperlipidemia. (**A**) Modeling, group administration, and indicator detection of experimental animals. (**B**) Body weight of mice in control and high-fat diet groups. (**C**) Food intake of mice in control and high-fat diet groups. (**D**) Representative images of the abdominal cavity and adipose area. (**E**) Weight of free abdominal adipose tissue and epididymal adipose tissue. (**F**) Changes in serum TC, TG, HDL-C, LDL-C, and Glu in mice. Data are expressed as mean ± SD. * *p* < 0.05, ** *p* < 0.01 vs. control group. The arrows indicate the experimental procedure.

We established models, managed group allocation, and conducted indicator measurements for experimental animals (Figure 1A). By analyzing the effects of SLKTE on high-fat-diet-induced obesity, the results demonstrated that after 8 weeks of feeding a high-fat diet, the body weight of mice in the experimental group was significantly higher than that of the control group (*p* < 0.05), as shown in Figure 1B. The food intake of mice in both the control group and the high-fat diet group is depicted in Figure 1C. Additionally, the weights of abdominal white fat and epididymal fat in the high-fat diet group were highly significantly increased (*p* < 0.01), as illustrated in Figure 1D,E. After 8 weeks of high-fat diet feeding, the serum levels of TC, TG, LDL-C, and Glu in the high-fat diet group were significantly higher than those in the control group, while the HDL-C level was significantly lower (*p* < 0.01), indicating the successful establishment of the hyperlipidemia model (Figure 1F).

**Figure 2:**
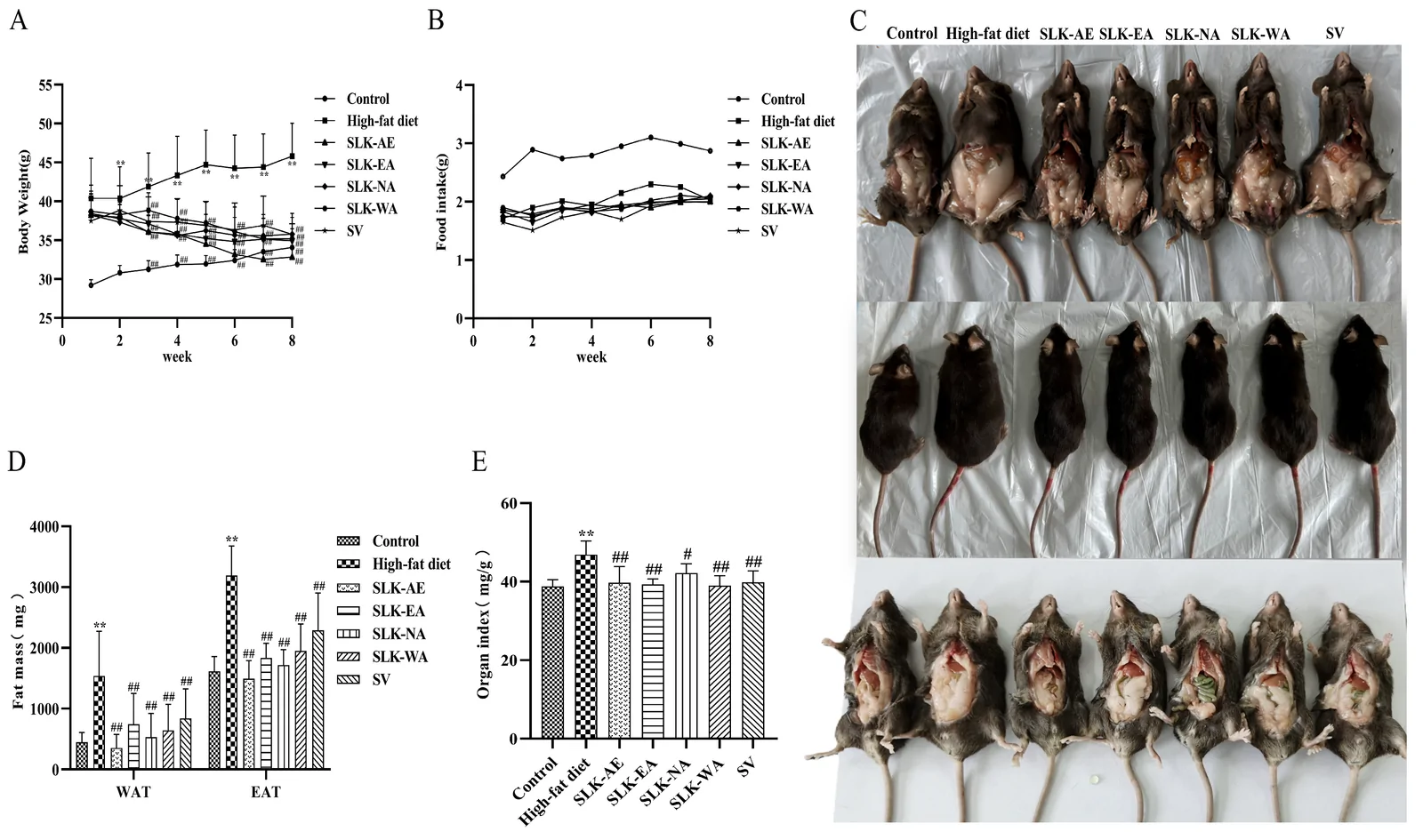
SLKTE attenuates high-fat-diet-induced obesity. (**A**) Body weights of seven groups of mice, including the control group, high-fat diet group, SLK-AE group, SLK-EA group, SLK-NA group, SLK-WA group, and SV group. (**B**) Food intake of mice in seven groups. (**C**) Representative images of the abdominal cavity and adipose area. (**D**) Weights of free abdominal adipose tissue and epididymal adipose tissue. (**E**) Organ index of liver tissue (mg/g). Data are expressed as mean ± SD. ** *p* < 0.01 vs. control group; # *p* < 0.05, ## *p* < 0.01 vs. HFD group.

Following 8 weeks of gavage treatment, the body weights of mice in all SLKTE treatment groups (SLK-AE, SLK-EA, SLK-NA, SLK-WA) and the SV group were significantly reduced compared to those in the high-fat diet group and were not significantly different from those of the control group (*p* < 0.01), as shown in Figure 2A. The food intake of mice in each group is presented in Figure 2B. Consistent with the trend of body weight reduction, the weights of abdominal white fat and epididymal fat in the SLK-AE, SLK-EA, SLK-NA, SLK-WA, and SV groups were also significantly reduced (*p* < 0.01), as shown in Figure 2C,D. Furthermore, the liver organ index (mg/g) of mice in the high-fat diet group was significantly higher compared to that in the control group (*p* < 0.01). After 8 weeks of administration, the liver organ index of mice in all treatment groups was significantly reduced (*p* < 0.05), as shown in Figure 2E. Thus, SLKTE significantly attenuated high-fat-diet-induced obesity and improved obesity-related biochemical indices, suggesting its potential anti-obesity effects.

### 3.3. Effect of SLKTE on Serum Lipid, Liver Injury, and Inflammation Levels in Hyperlipidemic Mice

**Figure 3:**
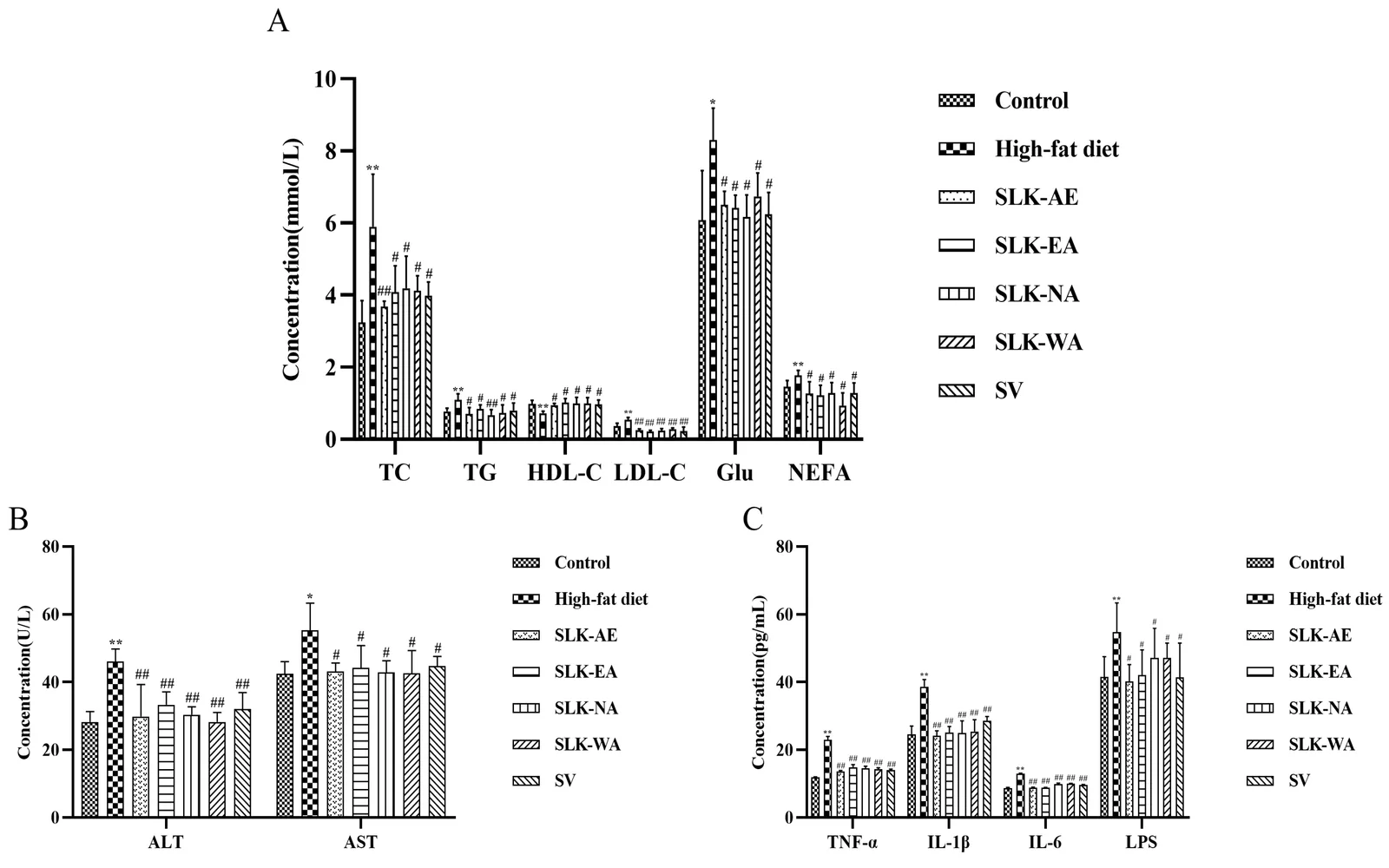
SLKTE improves serum levels of lipids, liver damage, and inflammation in hyperlipidemic mice. (**A**) Changes in serum TC, TG, HDL-C, LDL-C, Glu, and NEFA in seven groups of mice. (**B**) Serum ALT and AST levels in seven groups of mice. (**C**) Serum TNF-α, IL-1β, IL-6, and LPS levels in seven groups of mice. Data are expressed as mean ± SD. * *p* < 0.05, ** *p* < 0.01 vs. control group; # *p* < 0.05, ## *p* < 0.01 vs. HFD group.

By analyzing the effects of SLKTE on high-fat-diet-induced obesity, the results revealed that, compared to the high-fat diet group, the serum levels of TC, TG, LDL-C, Glu, and NEFA in all SLKTE groups (SLK-AE, SLK-EA, SLK-NA, and SLK-WA) and the SV group were significantly reduced (*p* < 0.05), while the HDL-C levels were significantly increased (*p* < 0.05), as shown in Figure 3A. Additionally, serum levels of ALT and AST were significantly elevated in the high-fat diet group compared to the control group (*p* < 0.05). In contrast, ALT levels were significantly reduced (*p* < 0.01) and AST levels were significantly reduced (*p* < 0.05) in all SLKTE groups and the SV group, demonstrating a therapeutic effect similar to that of the positive control drug, as shown in Figure 3B.

Regarding inflammation, the serum levels of TNF-α, IL-1β, IL-6, and LPS in the high-fat diet group were extremely significantly higher than those in the control group (*p* < 0.01). After 8 weeks of gavage treatment, these levels were significantly reduced in all SLKTE groups and the SV group (*p* < 0.05), as shown in Figure 3C. These results suggest potential anti-inflammatory effects of SLKT fractions in HFD-induced hyperlipidemic mice, as evidenced by alterations in circulating cytokine levels. However, it should be noted that these findings are based on serum cytokine measurements, and direct assessment of tissue inflammatory markers (e.g., hepatic macrophage infiltration, NF-κB activation) would be needed to confirm local anti-inflammatory actions. Thus, SLKTE significantly improved serum lipid levels, liver injury markers, and inflammatory factor levels in hyperlipidemic mice, suggesting its potential lipid-lowering, hepatoprotective, and anti-inflammatory effects.

### 3.4. Amelioration of Hepatic Lipids, Hepatic Injury, and Oxidative Stress Levels in Hyperlipidemic Mice by SLKTE

**Figure 4:**
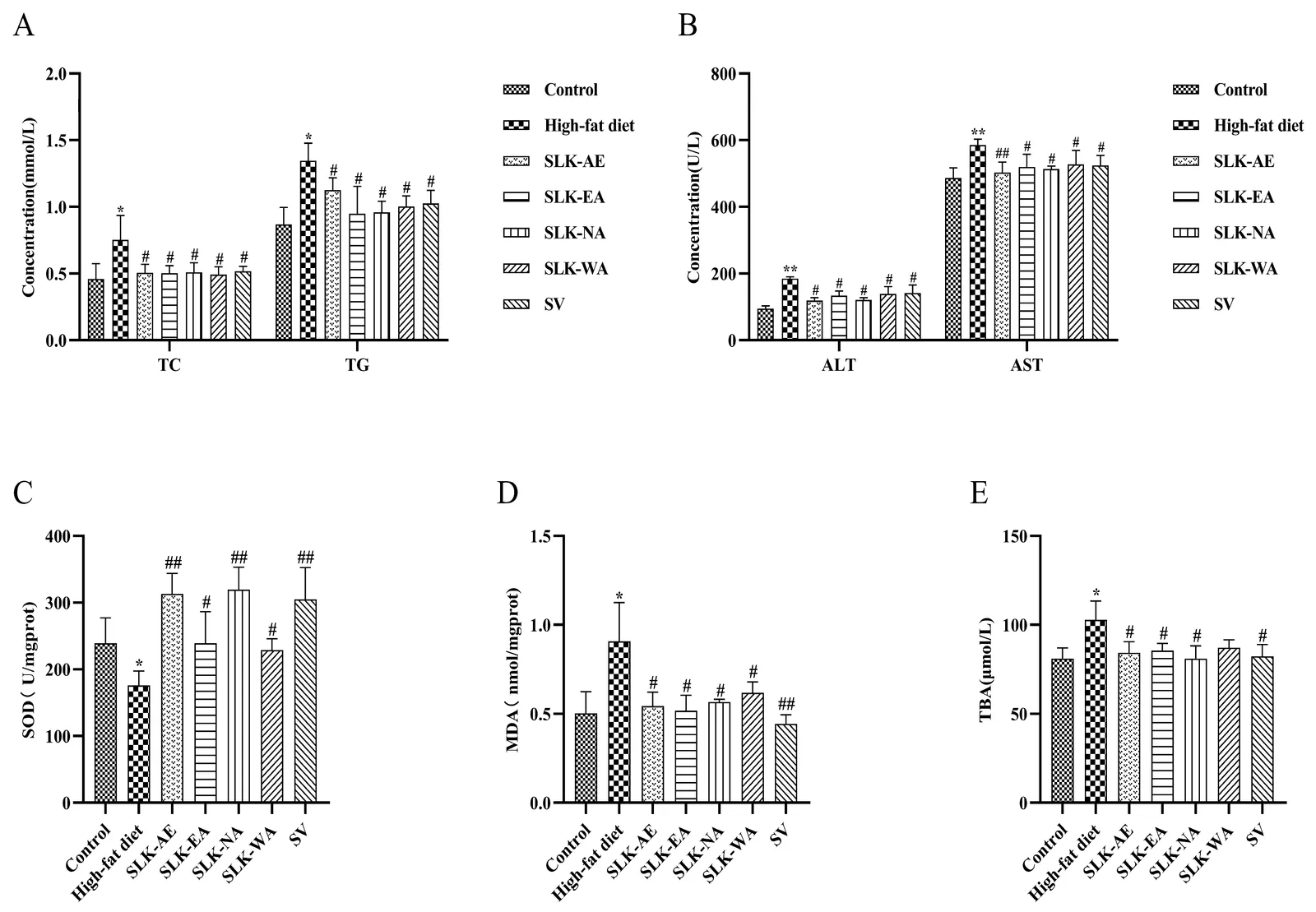
SLKTE improves lipid, liver damage, and oxidative stress levels in the liver of hyperlipidemic mice. (**A**) TC and TG levels in the liver of mice. (**B**) ALT and AST values in the liver of mice. (**C**) SOD levels in the liver of mice. (**D**) MDA levels in the liver of mice. (**E**) TBA levels in the liver of mice. Data are expressed as mean ± SD. * *p* < 0.05, ** *p* < 0.01 vs. control group; # *p* < 0.05, ## *p* < 0.01 vs. HFD group.

By analyzing the effects of SLKTE on hepatic lipid levels, liver injury, and oxidative stress in hyperlipidemic mice, the results indicated that the levels of TC and TG were significantly elevated in the liver tissues of mice fed a high-fat diet compared to the control group. In contrast, liver TC and TG levels were significantly lower (*p* < 0.05) in all SLKTE treatment groups (SLK-AE, SLK-EA, SLK-NA, SLK-WA) and the SV group compared to the high-fat diet group, as shown in Figure 4A. Additionally, the levels of ALT and AST were significantly elevated in the liver tissues of mice in the high-fat diet group compared to the control group (*p* < 0.01). However, ALT and AST levels were significantly reduced (*p* < 0.05) in all SLKTE groups and the SV group compared to the high-fat diet group, as shown in Figure 4B.

Regarding oxidative stress, SOD levels were significantly reduced in the liver tissues of mice in the high-fat diet group compared to the control group, while SOD levels were significantly increased (*p* < 0.05) after SLKTE administration, as shown in Figure 4C. Meanwhile, MDA levels were significantly increased in the liver tissues of mice in the high-fat diet group compared to the control group, but were significantly decreased (*p* < 0.05) after 8 weeks of SLKTE administration, as shown in Figure 4D. Compared to the control group, TBA levels in the liver tissues of mice in the high-fat diet group were significantly increased. TBA levels in the SLK-AE, SLK-EA, SLK-NA, and SV groups were significantly reduced (*p* < 0.05), while the reduction in the SLK-WA group was not statistically significant (*p* > 0.05), as shown in Figure 4E.

Thus, SLKTE significantly improved hepatic lipid levels, liver injury markers, and oxidative stress levels in hyperlipidemic mice, demonstrating its potential hepatoprotective effects in the treatment of hyperlipidemia.

### 3.5. SLKTE Attenuates Hepatic Steatosis Due to Hyperlipidemia

**Figure 5:**
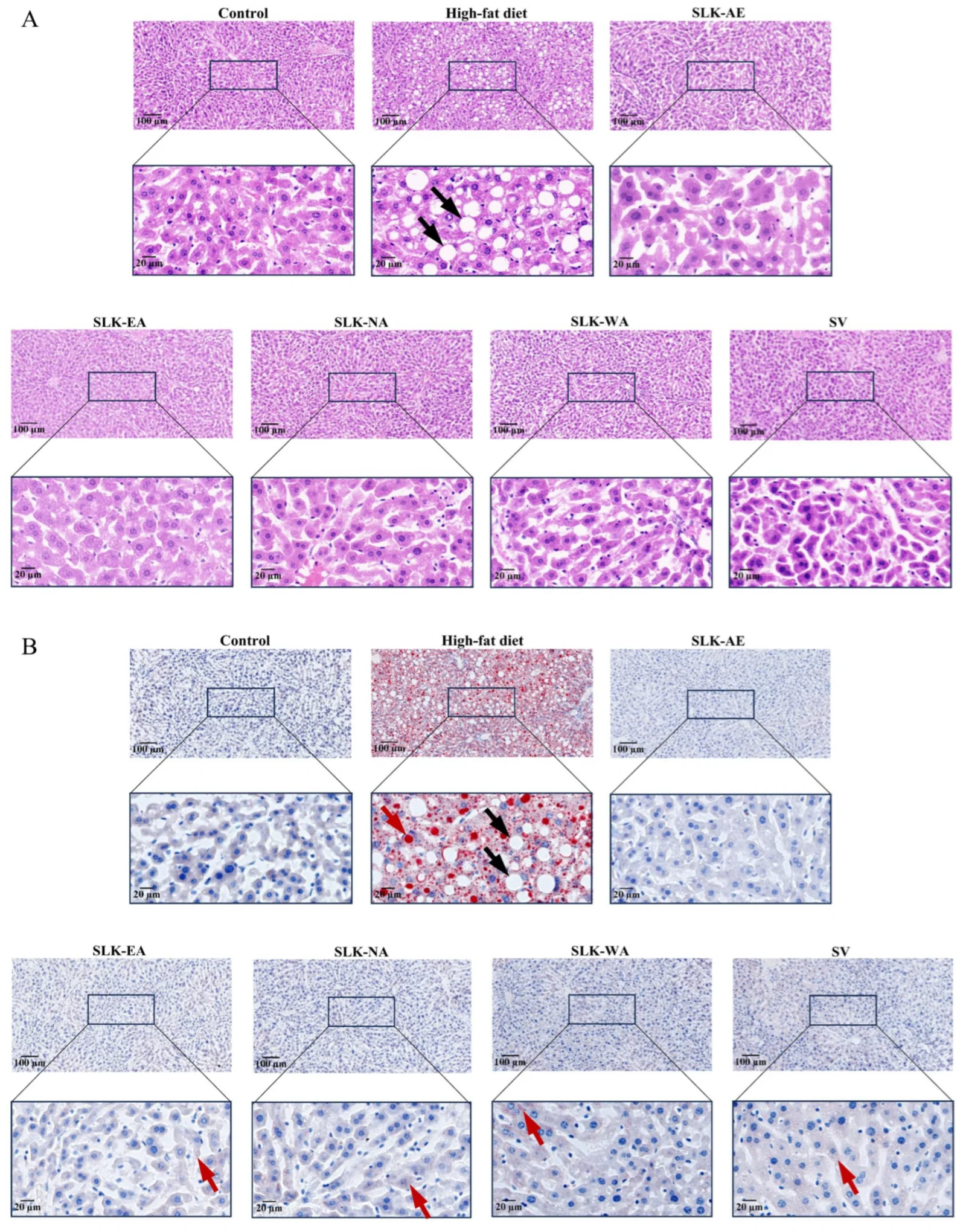
SLKTE rescues high-fat-diet-induced liver steatosis. (**A**) Representative images of H&E-stained liver sections. (**B**) Representative images of Oil Red O-stained liver sections. Scale bars, 100 μm. Zoom scale bars: 20 μm. Black arrows indicate typical foam cells with lipid-laden vacuolated cytoplasm, and red arrows indicate typical red lipid droplets.

By analyzing the effects of SLKTE on hyperlipidemia-induced hepatic steatosis, histological examination using H&E staining revealed that the liver tissue structure of the control group exhibited a normal, homogeneous appearance with no evident lipid droplet deposition or pathological abnormalities. In contrast, the hepatocyte nuclei in the high-fat diet group appeared lighter in color, suggesting potential hepatocyte damage, and numerous vacuolated lipid droplets were observed in the cytoplasm, indicating significant hepatic steatosis induced by the high-fat diet. After gavage treatment with SLKTE (SLK-AE, SLK-EA, SLK-NA, and SLK-WA groups) and the SV group, the lipid droplets in the hepatocytes of these mice were significantly reduced, effectively attenuating the degree of hepatic steatosis, as shown in Figure 5A.

Oil Red O staining further confirmed these findings. No obvious red lipid droplets were observed in the liver tissues of the control group, whereas the high-fat diet group exhibited many widely distributed red lipid droplets of varying sizes and round or oval morphology, reflecting hepatic lipid accumulation induced by the high-fat diet. In contrast, the number of lipid droplets was significantly reduced in all SLKTE groups and the SV group, with only a few small lipid droplets scattered throughout the tissue, as shown in Figure 5B. These results further confirmed the significant alleviating effect of SLKTE on hyperlipidemia-induced hepatic steatosis.

In conclusion, SLKTE effectively ameliorated hepatic steatosis in hyperlipidemic mice by reducing lipid droplet deposition in the liver, suggesting its potential as a therapeutic strategy for hyperlipidemia-associated hepatic pathological changes.

### 3.6. Effect of SLKTE on Liver ACADM, EHHADH, and HADHB Protein Levels in Hyperlipidemic Mice

The present study analyzed the effects of SLKTE on the protein levels of ACADM, EHHADH, and HADHB in the liver tissues of hyperlipidemic mice. ACADM catalyzes the initial dehydrogenation of medium-chain acyl-CoA substrates and is a rate-limiting enzyme in β-oxidation; EHHADH is a bifunctional peroxisomal enzyme that catalyzes both the hydration and the second dehydrogenation steps; HADHB is the beta subunit of the mitochondrial trifunctional protein, which catalyzes the final three steps of long-chain fatty acid β-oxidation. Together, these three enzymes cover the initiation, middle, and terminal stages of the β-oxidation cascade. Using the Western blot technique, the study quantified the protein expression levels of these key fatty acid oxidation enzymes. The results indicated that the protein levels of ACADM, EHHADH, and HADHB were significantly downregulated in the liver tissues of mice fed a high-fat diet compared to those fed a normal diet (*p* < 0.05). This finding suggests that a high-fat diet may disrupt normal lipid metabolism processes in the liver.

**Figure 6:**
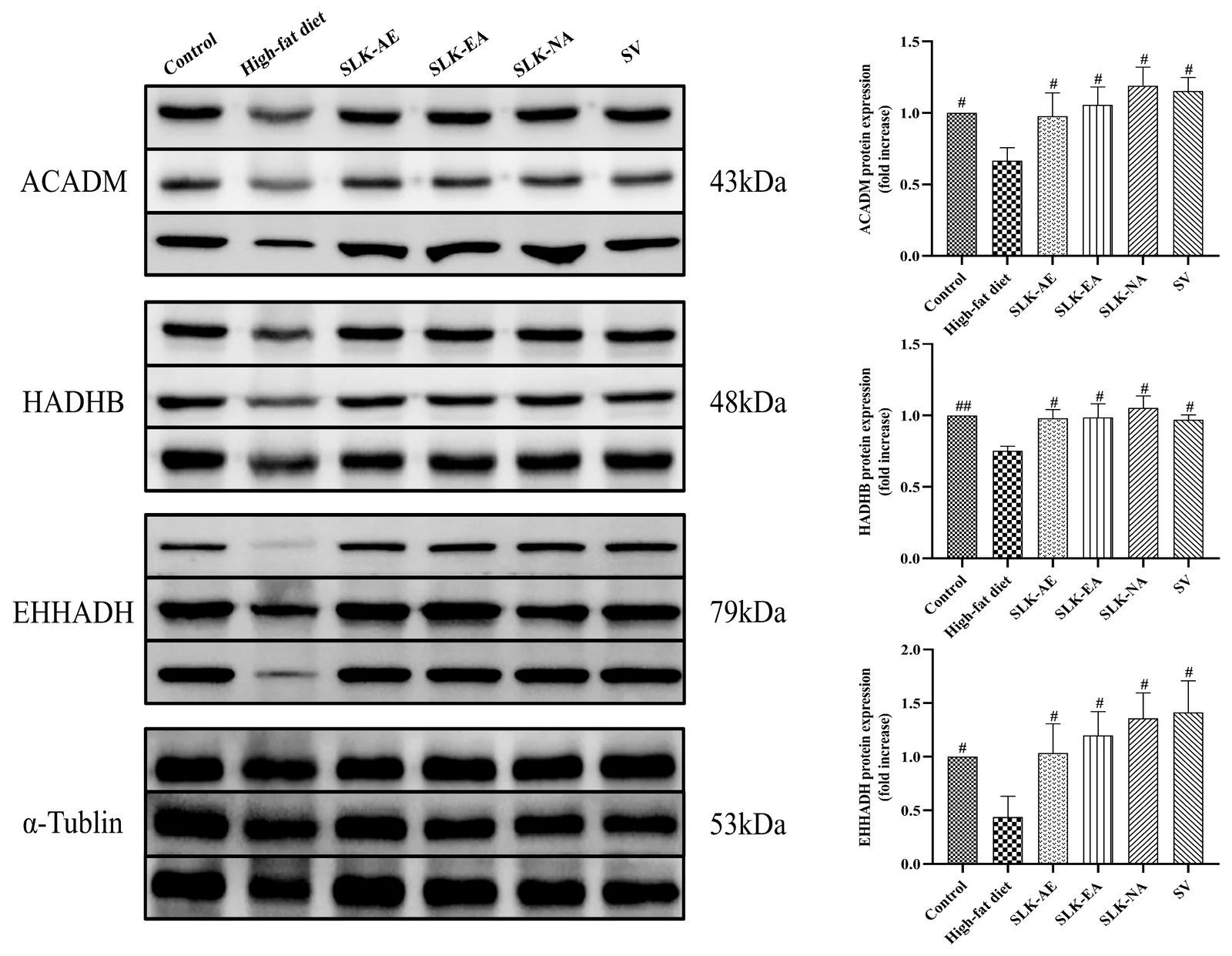
Effect of SLKTE on ACADM, EHHADH, and HADHB proteins in the liver of hyperlipidemic mice. Data are expressed as mean ± SD. # *p* < 0.05, ## *p* < 0.01 vs. HFD group.

Further analysis revealed that the expression levels of ACADM, EHHADH, and HADHB proteins were significantly upregulated (*p* < 0.05) in the liver tissues treated with SLKTE (SLK-AE, SLK-EA, SLK-NA groups) and the SV group compared to the high-fat diet group, as shown in Figure 6. These results indicate that SLKTE can effectively reverse the high-fat-diet-induced downregulation of key fatty acid oxidation enzymes in the liver. Consequently, by upregulating the expression of these enzymes, SLKTE may promote the oxidative degradation of fatty acids, thereby positively regulating lipid metabolism disorders in hyperlipidemic mice.

## 4. Discussion

Hyperlipidemia is a significant risk factor for cardiovascular diseases, characterized by abnormally elevated levels of TC, TG, and LDL-C, coupled with a decrease in HDL-C [24]. Prolonged hyperlipidemia can lead to vascular hardening, atherosclerosis, and other serious health complications, such as fatty liver disease and metabolic disorders. Therefore, this study aimed to investigate the effects of SLKTE on hyperlipidemic mice, focusing on its ability to improve lipid profiles, reduce liver damage, and regulate inflammation and oxidative stress. Additionally, the potential molecular mechanisms underlying these effects were explored.

The study demonstrated that SLKTE effectively inhibited body weight gain in hyperlipidemic mice, as evidenced by significant reductions in body weight, abdominal white fat, and epididymal fat weight. This suggests that SLKTE may improve fat metabolism or inhibit fat storage, thereby mitigating obesity-related complications [25]. SLKTE significantly reduced serum levels of TC, TG, LDL-C, Glu, and NEFA, while simultaneously increasing HDL-C levels. These findings indicate that SLKTE can effectively ameliorate lipid abnormalities in mice, reducing the risk of fat accumulation and potentially acting by inhibiting lipid synthesis or promoting lipid metabolism [26,27].

Chronic low-grade inflammation is a hallmark of obesity and metabolic diseases, with excessive release of inflammatory factors exacerbating metabolic disorders and further aggravating conditions such as obesity, insulin resistance, and fatty liver disease [28]. SLKT treatment was associated with reduced levels of lipid peroxidation markers (MDA) and pro-inflammatory cytokines (TNF-α, IL-1β, IL-6, LPS), suggesting potential anti-inflammatory and antioxidant activities. However, these findings are based on serum and hepatic homogenate measurements; direct assessment of tissue inflammatory markers (e.g., hepatic macrophage infiltration, NF-κB activation) and oxidative damage at the cellular level would be required to confirm local anti-inflammatory and antioxidant actions. SLKTE effectively alleviated oxidative damage and lipid peroxidation in liver cells, likely through the regulation of immune responses, reduction in fat accumulation, and mitigation of inflammation. Consequently, this improves the pathological state of hyperlipidemia [29]. The natural antioxidant components in SLKT, such as polyphenolic compounds (including phenolic acids and flavonoids), can protect liver cells by scavenging free radicals and reducing oxidative damage, ultimately lowering the release of ALT and AST [30,31]. Histological examinations using H&E staining and Oil Red O staining revealed a significant reduction in lipid droplets in the hepatocytes of the treatment group mice, effectively alleviating fatty degeneration in the cytoplasm [32]. This suggests that SLKTE may improve liver cell health by regulating lipid metabolism, promoting fat metabolism and decomposition, and reducing hepatic fat deposition [33].

The comparative analysis of the four SLKTE fractions revealed a clear structure–activity relationship. SLKTE demonstrated efficacy across all measured parameters, including serum lipid profiles, hepatic steatosis reduction, and upregulation of β-oxidation enzymes.

The petroleum ether fraction was generated as an intermediate defatting/degreasing product during sequential solvent partitioning in our previous study [23]. However, SLK-PE was not selected for in vivo testing, as the recovered sample mass was insufficient to support the full 8-week animal study at the planned experimental dose.

With respect to the potential hepatotoxicity of residual ethanol from the extraction process, we note that the anhydrous ethanol fraction (SLK-AE) underwent complete solvent removal through rotary evaporation followed by freeze-drying, yielding a solvent-free lyophilized powder. Furthermore, if residual solvent had affected liver function, we would expect elevated ALT/AST levels in the SLK-AE group; however, our results demonstrated significant reductions in these markers (Figure 3B and Figure 4B), confirming no solvent-related hepatotoxicity. One additional limitation should be highlighted: SLK-AE was prepared independently in the present study from a different starting-material batch relative to SLK-EA, SLK-NA and SLK-WA obtained from Ref. [23]. Future investigations are required to verify batch-to-batch chemical-composition consistency across these fractions.

ACADM, EHHADH, and HADHB are key enzymes in the fatty acid degradation pathway, playing essential roles in lipid metabolism and participating in the β-oxidation of fatty acids. These enzymes are particularly important in the liver, where they contribute to fatty acid degradation and energy production [34]. ACADM is a critical enzyme responsible for the β-oxidation of long-chain fatty acids and is involved in fatty acid catabolism. Under SLKTE treatment, the expression of ACADM was significantly upregulated, potentially enhancing fatty acid oxidation and reducing triglyceride accumulation in the blood [35]. EHHADH is another enzyme involved in the β-oxidation of fatty acids and plays a significant role in regulating lipid metabolism. The upregulation of EHHADH promotes fatty acid degradation, thereby alleviating fat accumulation in the liver and other tissues [36,37,38]. HADHB is also a key enzyme in fatty acid degradation, participating in the β-oxidation process and playing a crucial role in ketone body generation. Its upregulation further supports the mechanism by which SLKTE reduces lipid levels through improved fatty acid degradation [39]. The significant upregulation of the protein expression levels of ACADM, EHHADH, and HADHB in the treatment group indicates that SLKTE effectively promotes the synthesis and expression of lipid metabolism enzymes. The enhanced activity of these enzymes accelerates fatty acid oxidation and decomposition, thereby reducing hepatic fat deposition, improving lipid metabolism disorders, and alleviating fatty liver disease and other metabolic disorders induced by a high-fat diet [40].

The chemical profiling of the SLKTE fraction identified a rich array of flavonoids, phenylethanoid glycosides, and organic acids. Among them, rhoifolin, poliumoside, apigenin-7-O-β-D-glucoside, and rutin exhibited the largest peak areas, suggesting that these compounds may serve as the principal bioactive constituents responsible for the lipid-lowering effects of SLKT.

Accumulating evidence has demonstrated that rhoifolin exerts potent anti-inflammatory activity by suppressing the IKKβ/NF-κB signaling pathway, leading to reduced secretion of TNF-α, IL-1β, and IL-6 in LPS-stimulated RAW264.7 macrophages and in acute inflammation animal models [41]. Moreover, rhoifolin has been shown to regulate oxidative stress and proinflammatory cytokine levels in Freund’s adjuvant-induced rheumatoid arthritis via inhibition of NF-κB, accompanied by decreased oxidative stress markers and reduced expression of TNF-α, IL-1β, and IL-6 [42]. These anti-inflammatory and antioxidative actions are in good agreement with our observations of reduced inflammatory markers and improved liver function following SLKT treatment.

Rutin, another major flavonoid component, has been reported to activate the AMPK pathway, thereby upregulating the expression of PGC-1α, NRF1, Tfam, and CPT1 in skeletal muscle, which promotes mitochondrial biogenesis and fatty acid β-oxidation, ultimately counteracting HFD-induced obesity and lipid accumulation [43]. This mechanistic insight aligns closely with our finding that SLKT treatment significantly upregulated the hepatic protein expression of key fatty acid β-oxidation enzymes, namely ACADM, EHHADH, and HADHB. These results strongly suggest that rutin, along with related flavonoid glycosides, may be a critical driver of the enhanced fatty acid degradation pathway observed in our study.

Furthermore, poliumoside, a phenylethanoid glycoside, has been characterized by strong antioxidant activity, with the ability to scavenge free radicals and inhibit erythrocyte hemolysis in vitro [44]. Poliumoside has also been documented to possess anti-inflammatory and antioxidant properties [45]. Phenylethanoid glycosides, including poliumoside, are known to exert their antioxidant effects not only by direct radical scavenging but also by modulating intracellular signaling pathways, such as activation of the Nrf2-ARE system and inhibition of NF-κB and MAPK pathways [43]. It is therefore plausible that these diverse constituents act synergistically to mediate the antioxidative and metabolic regulatory effects of SLKT.

Despite the promising findings, several limitations of this study should be acknowledged. First, the study was conducted exclusively in male C57BL/6J mice, which limits the generalizability of the findings to female subjects. Sex differences in lipid metabolism and drug responses are well documented, and future studies should include both male and female mice to investigate potential sex-dependent effects. Second, although we observed upregulation of β-oxidation enzymes (ACADM, EHHADH, and HADHB) in SLKT-treated mice, the causal relationship between enzyme upregulation and the observed lipid-lowering effects was not directly established. The correlation between enzyme expression and metabolic improvements is suggestive but not definitive. Future studies should incorporate pathway-specific inhibitors (e.g., PPAR-α antagonists such as GW6471, or β-oxidation inhibitors such as etomoxir) to determine whether the beneficial effects are abrogated when β-oxidation is blocked, thereby establishing causality. Third, the anti-inflammatory and antioxidant conclusions were based solely on serum cytokine measurements and hepatic homogenate markers (SOD, MDA); direct assessment of tissue inflammatory markers (e.g., hepatic macrophage infiltration, NF-κB activation) and cellular oxidative damage would be needed to confirm local anti-inflammatory and antioxidant actions. Fourth, comprehensive toxicological evaluation of SLKT fractions was not performed. While no adverse effects were observed at the tested dosage (500 mg/kg/day), systematic toxicological studies at higher concentrations and longer durations would be necessary to establish a complete safety profile for potential clinical translation. Fifth, the chemical characterization was limited to targeted quantification of known compounds based on previous LC-MS/MS data; untargeted metabolomics approaches could potentially identify novel bioactive constituents. Sixth, the study used a single dose (500 mg/kg/day) without a dose–response design, which precludes determination of the optimal therapeutic dose and the minimum effective dose. Seventh, the lipid-lowering and hepatoprotective bioactivities of SLKT fractions are likely derived from synergistic actions of multiple phytochemical constituents. In the present work, we only tentatively proposed several major peaks (rhoifolin, poliumoside, apigenin-7-O-β-D-glucoside, and rutin) as candidate bioactive substances according to LC-MS/MS peak intensity, but we could not sufficiently narrow down or functionally validate the exact individual or combinatorial bioactive compounds responsible for the in vivo protective phenotype. Further bioactivity-guided fractionation and isolation are required to pinpoint key active molecules. Eighth, practical translational relevance regarding our administered dose remains insufficiently addressed. We have not calculated the extraction yields of each SLKT fraction, the corresponding raw small-leaved Kuding tea leaf mass equivalent to the given mouse dosage (500 mg/kg/day), nor performed human-equivalent dose conversion according to allometric scaling. These parameters are critical to bridge animal experimental results to real-world human consumption scenarios of Kuding tea and will be explored in follow-up investigations.

Based on these limitations, future research directions include: (1) conducting dose–response and sex-comparative studies to establish optimal dosing regimens and assess sex-specific effects; (2) employing pathway inhibitors and gene knockout models to establish causal mechanisms linking β-oxidation enzyme upregulation to lipid-lowering effects; (3) performing comprehensive toxicological assessments, including acute and sub-chronic toxicity studies; (4) using untargeted metabolomics and network pharmacology approaches to identify novel bioactive compounds and their molecular targets; (5) conducting time-series studies with multiple intermediate time points (2, 4, 6, and 8 weeks) to elucidate the temporal dynamics of SLKT action; (6) investigating the effects of SLKT fractions in more advanced disease models; (7) applying bioactivity-guided separation together with untargeted metabolomics and network pharmacology approaches to screen, identify, and validate individual or synergistic bioactive constituents from SLKT fractions; (8) calculating fraction extraction yields, raw tea-leaf equivalents for experimental animal doses, and performing allometric scaling to estimate human-equivalent doses for better translational interpretation.

## 5. Conclusions

In summary, the present study provides evidence that solvent fractions of small-leaved Kuding tea, particularly the aqueous extract (SLK-AE) and ethyl acetate fraction (SLK-EA), are associated with improvements in serum lipid profiles, reduction in hepatic steatosis, and modulation of inflammatory and oxidative stress biomarkers in high-fat-diet-induced mice. These beneficial effects are accompanied by increased protein expression of key fatty acid β-oxidation enzymes—ACADM, EHHADH, and HADHB—suggesting that enhanced fatty acid oxidation may contribute to the observed improvements in lipid metabolism. However, given the correlative nature of the evidence and the limitations of the study design (including the use of only male mice, single-dose administration, and lack of pathway-specific mechanistic validation), these findings should be interpreted as suggestive rather than definitive. Further mechanistic studies using pathway inhibitors, comprehensive toxicological evaluations, dose–response studies, and clinical investigations are needed before SLKT can be recommended for clinical use in the management of hyperlipidemia and associated metabolic disorders.

## Data Availability

Data can be shared from the corresponding author when required.

## Supplementary Materials

The following supporting information can be downloaded at https://www.mdpi.com/article/10.3390/nu18203345/s1, Table S1: Chemical constituents of SLK-AE extracts; Table S2: Chemometric analysis of the SLK-AE fraction—component profiling results.
