# Supplementary material for: Small-Leaved Kuding Tea Extract Fractions Attenuate Hyperlipidemia and Hepatic Steatosis in Mice: Association with Upregulation of Fatty Acid β-Oxidation Enzymes and Improvement of Metabolic Biomarkers
Source: Nutrients. 2026 Oct 13;18(20):3345. doi: 10.3390/nu18203345 (PMC13708154; doi:10.3390/nu18203345)
Supplement: Supplementary file 1 [file nutrients-18-03345-s001.zip › nutrients-4494585-supplementary.pdf]

Table S1 Chemical constituents of SLK-AE extracts.

anhydrous ethanol component (SLK-AE)		
3,4-Dihydroxyphenylethanol	Calceolarioside B	Guanosine
5-Hydroxy-1-tetralone	Coumarin	Hastatoside
5-Hydroxymethylfurfural	Cynaroside	Isoquercitrin
Adenine	Dacarbazine	Kaempferol
Adenosine	Dehydrodiisoeugenol	Kaempferol-3-O-rutinoside
Apigenin	Eriodictyol	Kaempferol-7-O-β-D-glucopyranoside
Apigenin-7-O-β-D-glucoside	Esculetin	Lactose
Atractyloside A	Forsythoside E	Lonicerin
Bilobalide	Grosvenorine	Luteolin
Caffeic acid	Guanine	Mannitol
Oleuropein	Prim-O-glucosylcimifugin	Rutin
Orsellinic acid	Protocatechualdehyde	Salidroside
p-Hydroxy-cinnamic acid	Purpureaside C	Skimmin
Plantamajoside	Quinic acid	Veraguensin
Poliumoside	Rhoifolin	Verbascoside
Verbenalin	Vicenin II	

Table S2 Chemometric analysis of the SLK-AE fraction — component profiling results

Number	Name	RT [min]	Formula	Molecular Weight	mzVault Best Match	Area: kudingcha.raw (F1)
1	3,4-Dihydroxyphenylethanol	3.942	C8 H10 O3	154.06186	91.4	2486015.247
2	5-Hydroxy-1-tetralone	14.835	C10 H10 O2	162.06783	56.7	494135.7433
3	5-Hydroxymethylfurfural	4.252	C6 H6 O3	126.03187	83.8	2717691.277
4	Adenine	0.898	C5 H5 N5	118.02798	88.1	16967410.03
5	Adenosine	1.369	C10 H13 N5 O4	267.09676	91.3	3624355.248
6	Apigenin	14.51	C15 H10 O5	270.05239	92.9	8026593.927
7	Apigenin-7-O-β-D-glucoside	14.971	C21 H20 O10	432.10579	93.7	80582396.09
8	Atractyloside A	16.744	C21 H36 O10	494.23579	80	1463498.81
9	Bilobalide	9.473	C15 H18 O8	326.09978	52.9	4476455.636
10	Caffeic acid	9.804	C9 H8 O4	180.04129	80	3409670.063
11	Calceolarioside B	14.705	C23 H26 O11	478.14699	89.8	3059208.07
12	Coumarin	16.573	C9 H6 O2	146.03664	90	53350928.93
13	Cynaroside	13.824	C21 H20 O11	448.10049	90.2	60270493.01
14	Dacarbazine	5.388	C6 H10 N6 O	182.09192	56.3	3415881.508
15	Dehydrodiisoeugenol	24.033	C20 H22 O4	326.15172	72.7	4990262.953
16	Eriodictyol	17.18	C15 H12 O6	288.06318	94.9	5544991.449
17	Esculetin	9.534	C9 H6 O4	178.02556	94.4	3871020.345
18	Forsythoside E	6.25	C20 H30 O12	462.17335	82.5	15462467.45
19	Grosvenorine	12.976	C33 H40 O19	740.21612	89.3	14128437.86

20	Guanine	1.474	C5 H5 N5 O	134.02279	81.3	2286627.388
21	Guanosine	1.473	C10 H13 N5 O5	283.09145	91.3	1648774.177
22	Hastatoside	10.069	C17 H24 O11	450.13679	73.1	3763335.283
23	Isoquercitrin	13.728	C21 H20 O12	464.09511	94.6	10938743.08
24	Kaempferol	12.975	C15 H10 O6	286.04726	90	2943874.416
25	Kaempferol-3-O-rutinoside	14.301	C27 H30 O15	594.15813	92.8	10494272.28
26	Kaempferol-7-O- β -D-glucopyranoside	14.933	C21 H20 O11	448.1006	85.7	55458854.58
27	Lactose	0.875	C12 H22 O11	388.12138	83.2	15228124.67
28	Lonicerin	13.522	C27 H30 O15	594.15839	92.2	29929020.93
29	Luteolin	13.826	C15 H10 O6	286.04726	90.8	5603982.533
30	Mannitol	0.865	C6 H14 O6	182.07813	87.2	71552586.48
31	Oleuropein	15.462	C25 H32 O13	540.18367	59.5	77430400.67
32	Orsellinic acid	7.575	C8 H8 O4	168.04228	83.9	3618535.33
33	p-Hydroxy-cinnamic acid	12.082	C9 H8 O3	164.04629	92.6	3553847.796
34	Plantamajoside	12.226	C29 H36 O16	640.19996	74.5	32489092.98
35	Poliumoside	14.066	C35 H46 O19	770.26332	91.2	372283771
36	Prim-O-glucosylcimifugin	9.167	C22 H28 O11	514.16566	52.6	1015884.068
37	Protocatechualdehyde	6.169	C7 H6 O3	138.03047	91	8917146.667
38	Purpureaside C	12.464	C35 H46 O20	786.25831	81.4	55485985.6
39	Quinic acid	0.907	C7 H12 O6	192.0625	93.4	85300716.15
40	Rhoifolin	14.51	C27 H30 O14	578.16373	89	124315672.9
41	Rutin	13.376	C27 H30 O16	610.15346	94.6	77956379.58
42	Salidroside	6.635	C14 H20 O7	300.1205	92.6	2574843.632
43	Skimmin	17.628	C15 H16 O8	324.08449	83.8	10920771.2
44	Veraguensin	29.003	C22 H28 O5	372.1935	90.2	77430400.67

45	Verbascoside	13.885	C29 H36 O15	624.20497	94.5	47060280.57
46	Verbenalin	11.219	C17 H24 O10	434.14207	82.2	53350928.93
47	Vicenin II	11.515	C27 H30 O15	594.15831	93.2	1463498.81
